# Altered S-nitrosylation of p53 is responsible for impaired antioxidant response in skeletal muscle during aging

**DOI:** 10.18632/aging.101139

**Published:** 2016-12-20

**Authors:** Sara Baldelli, Maria Rosa Ciriolo

**Affiliations:** ^1^ Università Telematica San Raffaele Roma, Rome, Italy; ^2^ Department of Biology, University of Rome ‘Tor Vergata’, Rome, Italy; ^3^ IRCCS San Raffaele ‘La Pisana’, Rome, Italy

**Keywords:** S-nitrosylation, antioxidant, sarcopenia, aging, atrophy

## Abstract

p53 transcriptional activity has been proposed to regulate both homeostasis and sarcopenia of skeletal muscle during aging. However, the exact molecular function of p53 remains to be clearly defined. We demonstrated a requirement of nuclear p53 S-nitrosylation in inducing a nitric oxide/PGC-1α-mediated antioxidant pathway in skeletal muscle. Importantly, mutant form of p53-DNA binding domain (C124S) did not undergo nuclear S-nitrosylation and failed in inducing the expression of antioxidant genes (i.e. SOD2 and GCLC). Moreover, we found that during aging the nuclear S-nitrosylation of p53 significantly declines in gastrocnemius/soleus leading to an impairment of redox homeostasis of skeletal muscle. We suggested that decreased level of nuclear neuronal nitric oxide synthase (nNOS)/Syntrophin complex, which we observed during aging, could be responsible for impaired nuclear S-nitrosylation. Taken together, our data indicate that altered S-nitrosylation of p53 during aging could be a contributing factor of sarcopenia condition and of other skeletal muscle pathologies associated with oxidative/nitrosative stress.

## INTRODUCTION

Sarcopenia is defined as the degenerative loss of skeletal muscle size and function that occurs during aging. This condition may be exacerbated by a decrease in physical activity, metabolic changes and oxidative stress [1, 2]. In fact, at the molecular level, increase of oxidative/nitrosative stress has been implicated in muscle wasting of aged skeletal muscle [3, 4].

Next to this, it is known in literature that p53 (transformation related protein 53, Trp53) transcription factor plays important roles during both myogenesis and sarcopenia of skeletal muscle [5-7]. Under unstressed conditions p53 cooperates with the myogenic regulatory factor MyoD (myogenic differentiation 1) to promote myogenesis by binding p53-response elements (p53-RE) on retinoblastoma (pRb) gene promoter, indicating that this transcriptional control may play an important role during myogenesis [6, 8, 9]. On the contrary, p53 has also been shown to induce atrophy/sarcopenia. In particular, upon genotoxic stress p53 binds to a highly conserved p53-RE on the Myogenin gene and transcriptionally represses its transcription, favoring muscle degeneration [10]. Despite such evidence, the molecular mechanisms responsible for the induction of p53 in myogenesis or muscle atrophy/sarcopenia remain to be clearly determined.

We previously highlighted a key role of p53 in regulating an antioxidant response essential for skeletal muscle homeostasis. Hence, under mild oxidative stress, p53 binds to the peroxisome proliferator-activated receptor gamma coactivator 1-alpha (PGC-1α) promoter, inducing the activation of a nitric oxide (NO)-dependent antioxidant signaling pathway [11]. Through this axis, p53 was able to buffer oxidative/nitrosative stress that otherwise would lead to premature sarcopenia and skeletal muscle atrophy. Moreover, we have recently demonstrated an impairment in the NO/PGC-1α-mediated signaling process in skeletal muscle of old mice, which resulted in increased oxidative/nitrosative stress [12].

In this work, we questioned whether the decline of the antioxidant response could be due to an alteration of p53 transcriptional activity on the *ppargc1a* promoter, resulting in increased oxidative/nitrosative stress and premature aging of skeletal muscle. We demonstrated that p53 S-nitrosylation at a specific cysteine residue (C124) is essential to assure efficient p53-mediated antioxidant response. Moreover, an altered shuttle of neuronal nitric oxide synthase (nNOS) to nuclear membrane during muscle aging is responsible for a decrement in nuclear S-nitrosylated p53. These findings clarify the role of NO in signaling transduction and provide evidence of its function in assuring a beneficial antioxidant signaling pathway in muscle tissue upon mild oxidative stress.

## RESULTS

### C124S mutation in p53-DBD causes inhibition of NO/PGC-1α-mediated antioxidant response

We have previously demonstrated that p53 was able to orchestrate a PGC-1α-mediated antioxidant response upon mild oxidative stress. Moreover, the inhibition of this signaling pathway results in increased levels of atrophy-related molecular factors in C2C12 myoblasts [11, 13]. Here, we deeply dissect the mechanism(s) through which p53 imposes pro-survival or pro-death pathway upon NO-mediated post-translational modifications of its DBD.

**Figure 1:**
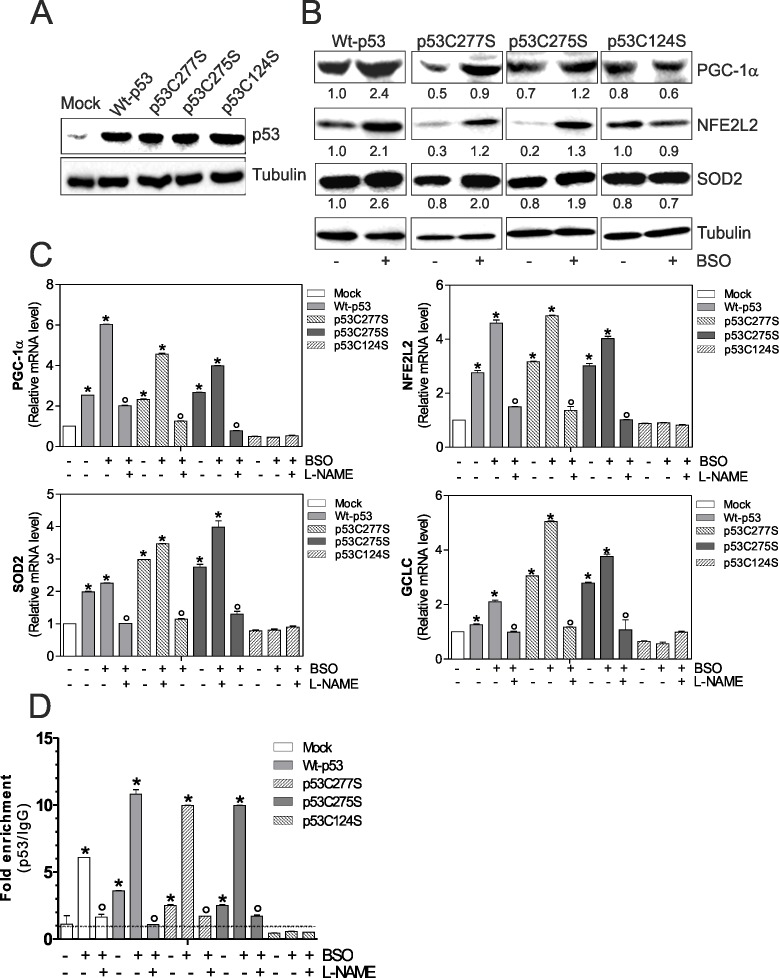
p53C124S mutant fails to induce NO/PGC-1α-mediated antioxidant pathway in C2C12 myoblasts (**A, B**) C2C12 myoblasts were transfected with pcDNA3.1 vector containing cDNA for wild type p53 (Wt-p53), three single p53 mutants in DBD (p53C277S, p53C275S, p53C124S) or with empty vector (Mock). After 15 h from transfection, myoblasts were treated with 1 mM BSO for 24 h. Cells were lysed and 20 μg of proteins were loaded for Western blot analysis of p53, PGC-1α, NFE2L2 and SOD2. Tubulin was used as loading control. Numbers indicate the density of immunoreactive bands calculated using the Software Quantity one (Bio-Rad) and reported as the ratio of PGC-1α, NFE2L2 and SOD2/Tubulin. (**C**) L-NAME (100 μM) was added 1 h before BSO treatment (15 h) and maintained throughout the experiment. Total RNA was isolated and relative mRNA levels of PGC-1α, NFE2L2, SOD2 and GCLC were analyzed by RT-qPCR. Data are expressed as means ± S.D. (n=4; *p<0.001 *vs* Mock; °p<0.001 *vs* BSO-treated cells). (**D**) ChIP assay was carried out on cross-linked nuclei from Mock, Wt-p53, p53C277S, p53C275S and p53C124S cells using p53 antibody followed by qPCR analysis of p53RE. Dashed line indicates the value of IgG control. Data are expressed as means ± S.D. (n=3; *p<0.001 *vs* Mock; °p<0.05 *vs* BSO-treated cells). All the immunoblots reported are from one experiment representative of four that gave similar results.

First, we transfected C2C12 myoblasts with either wild type p53 (Wt-p53) or single point mutation of DBD Cys277, 275 and 124 to Ser (p53C277S, p53C275S and p53C124S) (Fig.1A). As shown in Fig.1B an increase of PGC-1α protein level and its downstream antioxidant genes was observed in Wt-p53, p53C277S and p53C275S overexpressing myoblasts, upon 1mM BSO treatment. Contrarily, p53C124S mutant was completely unresponsive. A corresponding increase in the transcription levels of PGC-1α, NFE2L2 (Nuclear factor erythroid-derived 2-like 2), SOD2 (superoxide dismutase 2) and CGCL (Glutamate-cysteine ligase catalytic subunit) was also observed (Fig.1B and C).

To confirm the previously assessed involvement of NO in the induction of p53 we treated C2C12 myoblasts with a NOSs inhibitor, L-NAME, at the concentration of 100 μM. Fig.1C shows that L-NAME is able to prevent the increase of PGC-1α mRNA and its downstream target genes only in BSO-treated Mock, Wt-p53, p53C277S and p53C275S myoblasts.

It is known in literature that p53 is able to bind the −2317 p53RE on the mouse *ppargc1a* promoter, assuring the expression of PGC-1α [11]. Therefore, we evaluated whether the binding capacity of the mutant p53C124S could be affected. A 10-fold increase in the occupancy of −2317 p53RE in BSO-treated Wt-p53, -p53C277S and -p53C275S myoblasts was observed (Fig.1D). On the contrary, *ppargc1a* promoter was not bound by the mutant p53C124S (Fig.1D). Consistent with the role of NO in mediating PGC-1α expression, the binding of Wt-p53, p53C277S and p53C275S was efficiently reduced by L-NAME treatment (Fig.1D). These data suggest that only specific sequences of p53-DBD are capable to induce the *ppargc1a* gene transcription in a NO-dependent manner, contributing to the antioxidant response necessary for muscle homeostasis.

### S-nitrosylation of C124 promotes p53 binding on *ppargc1a* promoter

**Figure 2:**
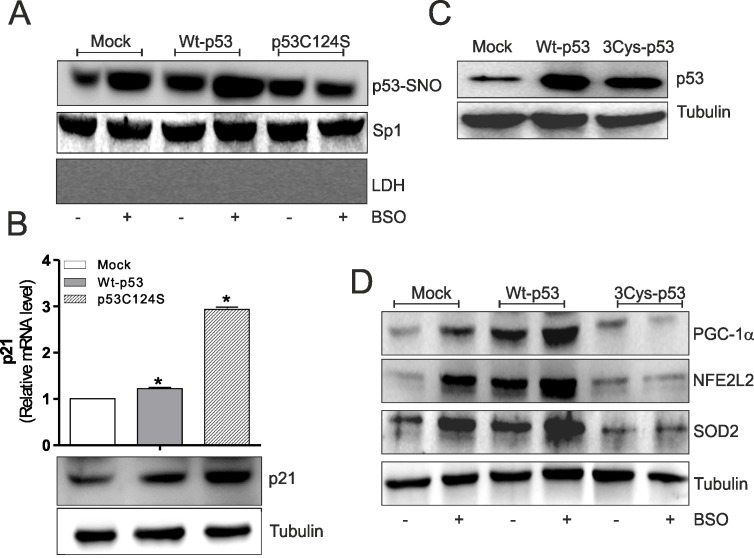
p53C124S mutant does not undergo S-nitrosylation after BSO treatment in C2C12 myoblasts (**A**) C2C12 myoblasts were transfected with pcDNA3.1 vector containing cDNA for wild type p53 (Wt-p53), single p53 mutant in DBD (p53C124S) or with empty vector (Mock). After 15 h from transfection myoblasts were treated with 1 mM BSO for 24 h. Nuclear proteins (500 μg) were subject to S-NO derivatization with biotin. After Western blot the nitrocellulose was incubated with p53 antibody for detection of p53-SNO. Sp1 was used as loading control. The possible presence of cytoplasmic contaminants was tested by incubating nitrocellulose with rabbit anti-LDH. (**B**) *Upper*: Total RNA was isolated and relative mRNA level of p21 was analyzed by RT-qPCR. Data are expressed as means ± S.D. (n=3; *p<0.05). *Bottom*: Cells were lysed and 20 μg of proteins were loaded for Western blot analysis of p21. Tubulin was used as loading control. (**C, D**) C2C12 myoblasts were transfected with pcDNA3.1 vector containing cDNA for wild type p53 (Wt-p53), triple p53 mutant in DBD (C277S, C275S and C124S) (3Cys-p53) or with empty vector (Mock). After 15 h from transfection myoblasts were treated with 1 mM BSO for 24 h. Cells were lysed and 20 μg of proteins were loaded for Western blot analysis of p53, PGC-1α, NFE2L2 and SOD2. Tubulin was used as loading control. All the immunoblots reported are from one experiment representative of five that gave similar results.

NO-mediated effects were primarily executed by protein S-nitrosylation, a reversible post-translational modification that produces NO-cysteine-thiol engagement [14]. S-nitrosylation represents an important post-translational modification that affects the functionality of p53, activating or inhibiting its binding on gene promoters [15, 16]. Thus, on the basis of results obtained, p53 S-nitrosylation status could be responsible for the ability to repress/activate *ppargc1a* gene transcription in skeletal muscle cells. Using the biotin-switch technique, we found that Wt-p53 myoblasts have increased level of S-nitrosylated p53 in nuclei (p53-SNO) than p53C124S myoblasts upon BSO treatment (Fig.2A). These results suggest that the increment of endogenous flux of NO due to GSH depletion [13], raises p53 transcriptional activity on *ppargc1a* promoter, through S-nitrosylation of a specific cysteine of its DBD. To clarify whether the low levels of nuclear p53C124S S-nitrosylation could affect the total transcriptional activity of p53 we analyzed the mRNA and protein levels of p21 (cyclin-dependent kinase inhibitor 1), a well-known p53-target gene. Fig.2B shows that mRNA (*upper*) and protein (*bottom*) levels of p21 were significantly higher in p53C124S myoblasts, demonstrating that the mutated form is transcriptionally active. The absence of S-nitrosylation on the specific C124 of p53-DBD mainly prevents the transcription of *ppargc1a* gene and the concomitant activation of antioxidant response in skeletal muscle.

The same set of experiments was performed using a triple p53-DBD mutant with the same three mutations Cys277, 275 and 124 to Ser (3Cys-p53) (Fig.2C). Western blot and RT-qPCR analysis of PGC-1α, NFE2L2, SOD2 and GCLC show that GSH deficiency increased their expression both in terms of protein (Fig.2D) and mRNA (Suppl.Fig.S1A) levels in Mock and Wt-p53 myoblasts. Contrarily, the transfection of 3Cys-p53 mutant failed to induce PGC-1α-mediated antioxidant pathway (Fig.2D and Suppl.Fig.S1A). Also, in this case, the inhibition of NOSs activity significantly reduced NO-mediated antioxidant pathway only in Mock and Wt-p53 cells (Suppl.Fig.S1A).

To investigate whether the binding capacity of 3Cys-p53 mutant on *ppargc1a* gene was affected, C2C12 nuclear extracts were incubated with biotinylated oligonucleotides representing −2317 p53RE and Western blot analysis of p53 was carried out after oligo pull-down. As reported in Suppl.Fig.S1B the p53 DNA-binding activity was significantly enhanced in BSO-treated Wt-p53 cells. Through quantitative ChIP analysis we also observed p53 enrichment at −2317 p53RE only in Wt-p53 myoblasts (Suppl.Fig.S1C) with respect to 3Cys-p53 mutant. In line with previous results, the concomitant administration of L-NAME completely abrogated the binding of p53 on *ppargc1a* promoter (Suppl.Fig.S1B and C). Moreover, using the biotin switch technique, we did not found any modulation of p53-SNO in nuclei of 3Cys-p53 myoblasts with respect Wt-p53 cells (Suppl.Fig.S2A). These results were also confirmed by evaluating Wt-p53 and 3Cys-p53 binding activity on *ppargc1a* promoter after treatment with a NO donor, S-nitrosoglutathione (GSNO). For this purpose we treated isolated nuclei from Wt-p53 and 3Cys-p53 myoblasts with GSNO and we performed an oligo-pull-down assay by using biotinylated oligonucleotides corresponding to −2317 p53RE. Suppl.Fig.S2B shows that the amount of Wt-p53 able to bind the −2317 p53RE was markedly higher only in GSNO-Wt-p53 treated nuclei and L-NAME treatment was able to restore this event. Contrarily, the use of NO scavenger carboxy-PTIO was not able to reduce the Wt-p53-binding on *ppargc1a* promoter after GSNO treatment (Suppl.Fig.S2B).

In summary, these data suggest that S-nitrosylation of p53 increases its transcriptional activity on *ppargc1a* gene after GSH depletion activating the antioxidants and ensuring the skeletal muscle homeostasis.

### S-nitrosylation of C124 is involved in p53 binding on PPARGC1A promoter also in p53-null-NCI-H1299 cell

**Figure 3:**
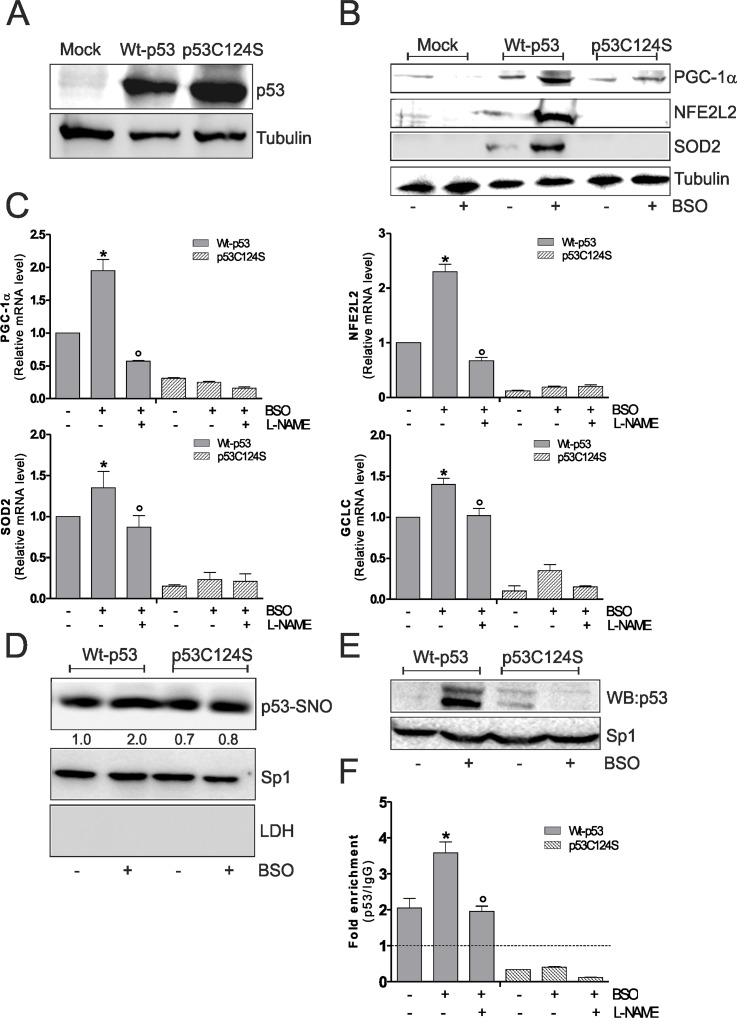
NO/PGC-1α-mediated antioxidant pathway is inhibited after p53C124S overexpression in p53-null NCI-H1299 cells (**A, B**) NCI-H1299 cells were transfected with pcDNA3.1 vector containing cDNA for wild type p53 (Wt-p53), single p53 mutant in DBD (p53C124S) or with empty vector (Mock). Cells were lysed and 20 μg of proteins were loaded for Western blot analysis of p53, PGC-1α, NFE2L2 and SOD2. Tubulin was used as loading control. (**C**) L-NAME (100 μM) was added 1 h before BSO treatment (15 h) and maintained throughout the experiment. Total RNA was isolated and relative mRNA levels of PGC-1α, NFE2L2, SOD2 and GCLC were analyzed by RT-qPCR. Data are expressed as means ± S.D. (n=3; *p<0.05 *vs* untreated Wt-p53; °p<0.001 *vs* BSO-treated Wt-p53 cells). (**D**) Nuclear proteins (500 μg) were subject to S-NO derivatization with biotin. After Western blot the nitrocellulose was incubated with p53 antibody for detection of p53-SNO. Sp1 was used as loading control. The possible presence of cytoplasmic contaminants was tested by incubating nitrocellulose with rabbit anti-LDH. Numbers indicate the density of immunoreactive bands calculated using the Software Quantity one (Bio-Rad) and reported as the ratio of p53-SNO/Sp1. (**E**) Nuclear protein extracts (500 μg) were subjected to oligo-pull-down by using the biotinylated oligonucleotide representing the p53RE on the PPARGC1A promoter and bound p53 was detected by Western blot. Twenty μg of nuclear proteins (input) were used for Western blot analysis of Sp1. (**F**) ChIP assay was carried out on cross-linked nuclei from Wt-p53 and p53C124S NCI-H1299 cells using p53 antibody followed by qPCR analysis of p53RE. Dashed line indicates the value of IgG control. Data are expressed as means ± S.D. (n=4; *p<0.05 *vs* untreated Wt-p53; °p<0.05 *vs* BSO-treated Wt-p53 cells). All the immunoblots reported are from one experiment representative of four that gave similar results.

Overall these results were confirmed in human lung cancer NCI-H1299 cells, which are null for p53. BSO treatment in these cells was able to induce PGC-1α, NFE2L2, SOD2 and GCLC up-regulation only after Wt-p53 transfection and L-NAME is able to inhibit the activation of this antioxidant pathway (Fig.3A, B and C). In accordance with previous results, Wt-p53 NCI-H1299 cells showed a significant increment of nuclear p53-SNO after BSO administration (Fig.3D). The capacity of Wt-p53 to bind the human PPARGC1A promoter at −1237 position was also investigated in NCI-H1299 cells. Fig.3E and F show the inability of p53C124S mutant to bind the PPARGC1A promoter upon GSH depletion. Conversely, an increase of PPARGC1A promoter occupancy in Wt-p53 cells was observed after BSO treatment, confirming the importance of a preserved p53-DBD and of p53 S-nitrosylation status in the signaling pathway that leads to NO/PGC-1α-mediated antioxidant response.

### C124S mutation induces atrophy in C2C12 myoblasts

**Figure 4:**
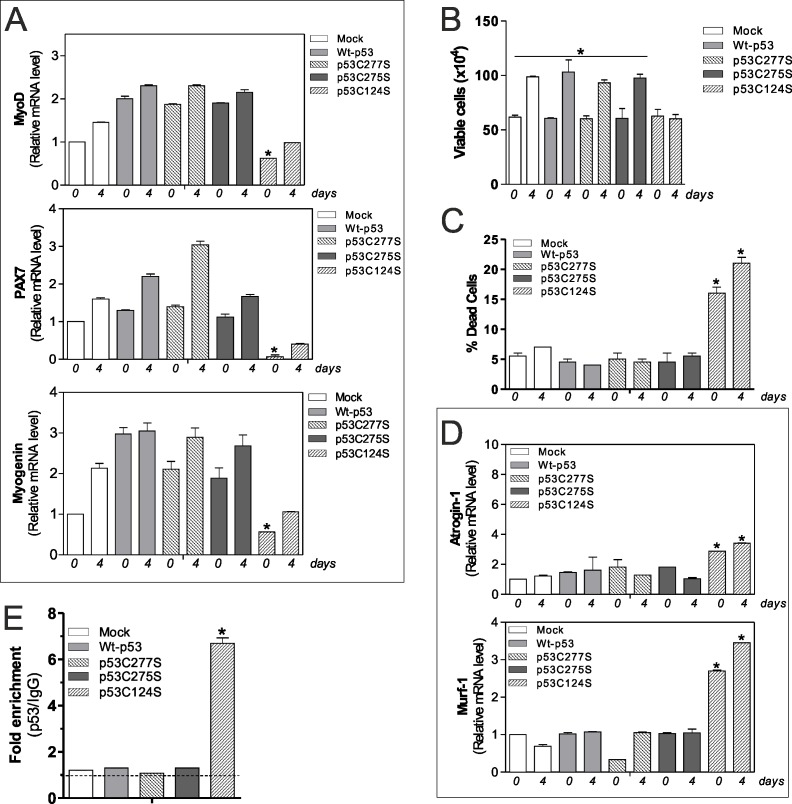
p53C124S mutant induces atrophy of C2C12 myoblasts (**A**) C2C12 myoblasts were transfected with pcDNA3.1 vector containing cDNA for wild type p53 (Wt-p53), three single p53 mutants in DBD (p53C277S, p53C275S, p53C124S) or with empty vector (Mock). After 24 h from transfection C2C12 cells were differentiated for 4 days. Total RNA was isolated and relative mRNA levels of MyoD, PAX7 and Myogenin were analyzed by RT-qPCR. Data are expressed as means ± S.D. All the values were significantly different with respect to Mock day 0/4 (n=3, p<0.05; *p<0.05 was significantly decreased with respect Mock day 0). (**B**) Cells were counted by Trypan Blue exclusion. Data are expressed as means ± S.D. All the values were significantly different with respect to day 0 (n=4 *p<0.05). (**C**) Dead cells were counted by Trypan blue exclusion. Data are expressed as means ± SD (n=4, *p<0.001 *vs* Mock-, Wt-p53-, p53C277S- and p53C275S-day 0/4 cells). (**D**) Total RNA was isolated and relative mRNA levels of MuRF-1 and Atrogin-1 were analyzed by RT-qPCR. Data are expressed as means ± S.D. (n=3; *p<0.05 *vs* Mock-, Wt-p53-, p53C277S- and p53C275S-day 0/4 cells). (**E**) ChIP assay was carried out on cross-linked nuclei from Mock, Wt-p53, p53C277S, p53C275S and p53C124S cells at day 4 of myogenesis using p53 antibody followed by qPCR analysis of p53RE. Dashed line indicates the value of IgG control. Data are expressed as means ± S.D. (n=3; *p<0.001 *vs* Mock-, Wt-p53-, p53C277S- and p53C275S-day 4 cells).

As mentioned above, p53C124S mutant interferes with antioxidant pathways in C2C12 myoblasts, probably altering the homeostasis of skeletal muscle. For this reason, we explored the effect of p53 single mutants on myoblasts differentiation program. We induced differentiation of C2C12 cells after transfection of Wt-p53, p53C277S p53C275S and p53C124S plasmids. Consistent with our previous results, cells transfected with Wt-p53, p53C277S, p53C275S and treated with differentiation medium exhibited increased mRNA levels of differentiation markers MyoD, Pax7 (Paired box 7) and Myogenin. On the contrary, p53C124S cells failed to increase such markers, suggesting a defective myogenesis (Fig.4A).

The effect of p53C124S overexpression on cell growth and viability was analyzed by direct counting by Trypan Blue staining. We found that the C124S mutation profoundly affected cell proliferation (Fig.4B) with an increase in dead cells (Fig.4C), indicating that both proliferation arrest and death occur. This is in line with the p21 protein increase observed in Fig.2B. Next, we have analyzed the mRNA levels of two atrophy markers Atrogin-1 (F-box protein 32) and MuRF-1 (tripartite motif-containing 63), which are two muscle-specific E3 ubiquitin ligases that are increased transcriptionally in skeletal muscle under atrophy-inducing conditions. Fig.4D highlights a significant raise of their expression only in undifferentiated and differentiated p53C124S cells, suggesting that this mutation not only inhibits antioxidant pathway but also blocks myoblasts cell differentiation inducing a degenerative process. To support the hypothesis that p53C124S mutant promotes the transcription of atrophy genes, we analyzed mouse MuRF-1 and Atrogin-1 promoters using Genomatix Software Suite database to identify p53RE. We have found five and one p53RE in mouse Atrogin-1 and MuRF-1 promoters, located at −519, −319, −82, −40, +31 and −351 respectively (Suppl.Fig.S2D, Suppl. Table S1). ChIP analyses of all p53RE were carried out to confirm the regulatory role of p53C124 mutant on the murine Atrogin-1 and MuRF-1 promoters. The qPCR analysis shows a significant increase only in p53C124S occupancy of −351 region on MuRF-1 promoter (Fig.4E), while −519, −319, −82, −40 and +31 regions on Atrogin-1 promoter did not show p53C124S binding at day 0 and 4 of differentiation (data not shown).

### Nuclear p53-S-nitrosylation diminished in aged skeletal muscle inhibiting antioxidant response

**Figure 5:**
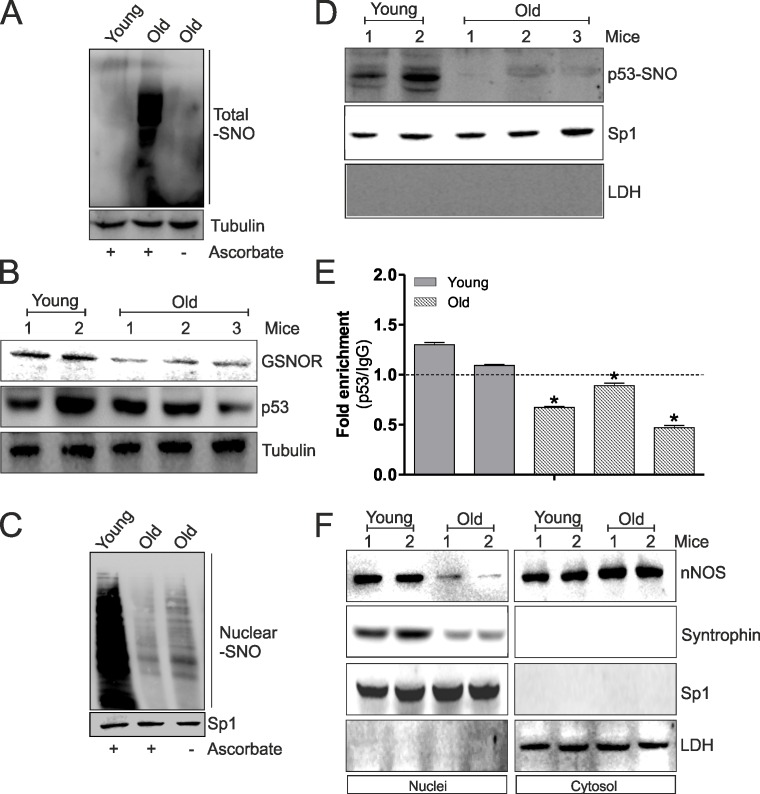
The decrement of nNOS nuclear localization inhibits p53 S-nitrosylation and its binding on *ppargc1a* promoter during aging (**A**) Skeletal muscle of young (12 weeks) and old (80 weeks) mice was homogenized and total proteins (500 μg) were subjected to S-NO derivatization with biotin. After Western blot, biotin adducts were identified by incubating nitrocellulose membrane with HRP-conjugate streptavidin. Proteins incubated in labeling buffer without ascorbate were used as negative control (−Ascorbate). Tubulin was used as loading control. (**B**) Skeletal muscle of two young (12 weeks) and three old (80 weeks) mice was homogenized and total proteins (20 μg) were loaded for Western blot analysis of GSNOR and p53. Tubulin was used as loading control. (**C**) Skeletal muscle of young (12 weeks) and old (80 weeks) mice was homogenized and nuclear proteins (500 μg) were subjected to S-NO derivatization with biotin. After Western blot, biotin adducts were identified by incubating nitrocellulose membrane with HRP-conjugate streptavidin. Proteins incubated in labeling buffer without ascorbate were used as negative control (−Ascorbate). Sp1 was used as loading control. (**D**) Skeletal muscle of two young (12 weeks) and three old (80 weeks) mice was homogenized and nuclear proteins (500 μg) were subjected to S-NO derivatization with biotin. After Western blot the nitrocellulose was incubated with p53 antibody for detection of p53-SNO. Sp1 was used as loading control. The possible presence of cytoplasmic contaminants was tested by incubating nitrocellulose with rabbit anti-LDH. (**E**) ChIP assay was carried out on cross-linked nuclei from two young (12 weeks) and three old (80 weeks) mice using p53 antibody followed by qPCR analysis of p53RE. Dashed line indicates the value of IgG control. Data are expressed as means ± S.D. (n=3; *p<0.05). (**F**) Skeletal muscle of two young (12 weeks) and two old (80 weeks) mice was homogenized and 20 μg of nuclear and cytoplasmic extracts were loaded for detection of nNOS and Syntrophin by Western blot. Sp1 was used as loading control. The possible presence of cytoplasmic contaminants was tested by incubating nitrocellulose with rabbit anti-LDH. All the immunoblots reported are from one experiment representative of four that gave similar results.

We have recently demonstrated that during aging of skeletal muscle a significant increase of oxidative/nitrosative stress occurs. In particular, we observed a decrease of GSH levels and a diminished induction of NO/PGC-1α-mediated redox signaling pathway accompanied by an accumulation of total S-nitrosylated proteins [12]. Since S-nitrosylation of C124 on p53-DBD seems to play a key role in the induction of NO/PGC-1α-mediated antioxidant pathway, we investigated the involvement of such mechanism in PGC-1α-mediated antioxidant response decrement during aging. Firstly, we confirmed the increase of total S-nitrosylated protein in skeletal muscle of old mice (Fig.5A). This result was also confirmed by the analysis of NADH-dependent *S*-nitrosoglutathione (GSNO) reductase (GSNOR), a denitrosylating enzyme that indirectly buffers the concentration of protein SNOs, by reducing the low-molecular-weight nitrosothiol GSNO. Fig.5B clearly indicates diminished protein levels of GSNOR in aged skeletal muscle, which could be in part responsible for S-nitrosylation protein increase. Moreover, total protein levels of p53 were slightly decreased in old with respect to young mice (Fig.5B). Subsequently, we analyzed the levels of nuclear S-nitrosylated proteins in aged skeletal muscle. As shown in Fig.5C, a decrease of nuclear S-nitrosylation is observed in skeletal muscle of aged mice. These results were confirmed by a decrease of p53-SNO levels in nuclei and a diminished binding of p53 on *ppargc1a* promoter (Fig.5D and E). These events probably elicited decreased PGC-1α-mediated antioxidant response observed during aging. Next to this, we have previously demonstrated that nNOS, interacting with Syntrophin, locates on nuclear membrane favoring local NO production, nuclear S-nitrosylation and induction of mitochondrial biogenesis during myogenesis [17]. These results strongly suggest the importance of nuclear nNOS activity in transcriptional regulation of NO/PGC-1α-mediated antioxidant target genes also during aging. For this reason, we analyzed nNOS and Syntrophin nuclear and cytoplasmic localization in young and old skeletal muscle. Western blot analysis of nNOS and Syntrophin carried out on nuclear fraction showed that these proteins were able to localize in nuclei only in young mice (Fig.5F), which exhibited a higher extent of S-nitrosylated proteins in nuclei with respect to aged mice (Fig.5C). Contrarily, nuclear associated nNOS was significantly diminished in aged mice, whereas its content is likely increased in cytoplasmic extracts (Fig.5F). Taken together, these results indicate that p53 binding on *ppargc1a* promoter requires the nuclear NO flux, which permits the transcription of *ppargc1a* gene and consequently the induction of antioxidant response, through the nitrosylation of p53-DBDC124. The decrement of nuclear nNOS localization during aging determines a loss of nuclear S-nitrosylated p53 and a failure in inducing PGC-1α-mediated pathway.

## DISCUSSION

Among the multifactorial aspects characterizing aging, sarcopenia, a continuous and progressive loss of muscle mass, represents an important public health problem related to frailty and disability [2]. Sarcopenia is characterized by alteration of a multitude of pathways largely ascribed to endocrine system and molecular processes that lead to the tight-related alterations in grip strength, physical performance and muscle mass homeostasis. Moreover, age-related changes in skeletal muscle are attributed also to activation and/or repression of specific molecules, such as atrogenes and PGC-1α [18, 19].

At molecular level a well-established hallmark of aged muscle is the increase in oxidative/nitrosative damage, which is recognized to contribute to skeletal muscle degeneration [20, 21]. Moreover, we found that, upon mild oxidative stress, nNOS is recruited to nuclei where it increases local NO production, S-nitrosylation of nuclear proteins and the induction of mitochondrial biogenesis in C2C12 cells [17]. In this context, p53 was capable to restrain oxidative stress, by orchestrating an antioxidant response through induction of antioxidant genes [11]. Overall data uncover an additional role for p53 in transcriptional regulation of genes involved in skeletal muscle homeostasis and functionality.

Here, we deeply dissected the p53-mediated signaling process under mild oxidative stress and we were able to identify a cysteine residue in the p53 DBD essential for the antioxidant response activation. Indeed, the data obtained with p53 mutants clearly indicated that the cysteine 124 was the sole and pivotal for induction of p53-mediated antioxidant response. Actually, the p53 C124S mutant failed to bind the p53RE on *ppargc1a* promoter abolishing the activation of antioxidant response.

Cysteine-S-nitrosylation represents one of the post-translational modifications through which redox signaling pathways can be tuned. Therefore, being aware that NO is the most suited molecule for constraining such modification and that upon mild redox unbalance its concentration raises at nuclear level, the data obtained with the C124S mutant undoubtedly indicated that this cysteine residue was prone to S-nitrosylation and this process was necessary for activating p53-downstream target genes. Even other cysteine residues characterized the p53 DBD domain, but only the C124S mutant did not undergo S-nitrosylation abolishing the binding to the *ppagc1a* promoter, highlighting, at the molecular level, the specificity of this residue in the redox signaling process.

We recently showed that during aging the increase of oxidative damage to proteins was paralleled by a decrement in glutathione, the main low molecular weight antioxidant, and complemented by the alteration of a PPAR-α/PGC-1α-mediated antioxidant signaling axis in gastrocnemius/soleus of old mice [4]. In this report, we demonstrated that although an increase in total protein S-nitrosylation occurred during muscle aging, a decrease of such process at nuclear level was observed. This evidence nicely associated with a concomitant decrement in p53 S-nitrosylation and with the observed diminished recruitment of nNOS/Syntrophin complex on nuclear membrane. We speculated that this failure by preventing nuclear sited NO flux inhibited local S-nitrosylation of p53 and might be responsible for the loss of efficient p53-SNO-dependent antioxidant response during aging. On the contrary, the increased total protein S-nitrosylation, in aged muscle, found a rationale in the observed glutathione decrement [4] as well as on the present data indicating a significant diminution of the expression levels of GSNOR. In fact, GSNOR is the primary system of the cell for degrading the main non-protein S-nitrosothiol GSNO, which by being in equilibrium with protein S-NOs, indirectly controls cellular concentrations of protein S-NOs [22]. The decrement in both glutathione and GSNOR makes the physiological NO flux more detrimental for proteins at cytosolic and other compartments, unless nucleus; another oxidative risk damage for aged muscle. Overall this evidence confirmed that NO is the principal mediator of p53 transcriptional activity on *ppargc1a* promoter. Accordingly, in-batch treatment of isolated nuclei with NO donor significantly increases the binding capacity of WT-p53 on its *consensus* sequence.

p53 plays important roles during differentiation and sarcopenia of skeletal muscle, though the exact mechanism(s) that regulate its transcriptional activity are still unclear. Some evidence demonstrated that the protein levels of p53 as well of p21 and GADD45a, two established transcriptional targets of p53, are higher in older muscle tissue, suggesting a requirement of p53 in promoting and regulating sarcopenia of skeletal muscle [23]. Schwarzkopf and colleagues suggested that chronic activation of p53 leads to premature aging associated with a significant atrophy [7, 24]. Contrarily, Feng *et al*. demonstrated a progressive decline of p53 protein level and p53-dependent pathways in various tissues of older mice, leading to premature aging [25]. Here, we give the proof that the inhibition of p53-SNO/PGC-1α-mediated antioxidant pathway is associated with increased markers of myotube degeneration. In particular, our data showed that failure of C124 S-nitrosylation switched the binding of p53 on MuRF-1 gene promoter inducing premature atrophy and cell death of skeletal muscle cells. Similarly, other studies reported that p53 knockout mice showed an alteration of mitochondrial activity in mixed muscle and lowered PGC-1ɑ protein levels in gastrocnemius. Intermyofibrillar mitochondria of these animals were characterized by reduced respiration and elevated reactive oxygen species production. In addition, these animals displayed greater fatigability and less locomotor endurance [26].

It is possible to postulate that C124-S-nitrosilatyion of p53 by inducing myogenesis and antioxidant response is implicated in maintaining skeletal muscle homeostasis and functions. Thus, our findings could be helpful for the comprehension of molecular mechanism underlying muscular pathologies or myopathies characterized by alteration in antioxidant response.

In conclusion, our results show that under mild oxidative stress nuclear NO is the primary mediator of p53 post-translational modification in skeletal muscle. S-nitrosylation of C124 is the mandatory event for NO/p53/PGC-1α-mediated antioxidant response. Thus, during aging the loss of nuclear nNOS/Syntrophin complex located on nuclear membrane may contribute to both an increase of total protein S-nitrosylation and myopathy. Therefore, maintaining/restoring p53 S-nitrosylation status could represent a new tool to prevent or treat myopathies and atrophy condition.

## METHODS

### Animals

We conducted all mouse experimentations in accordance with accepted standard of humane animal care and with the approval by relevant national (Ministry of Welfare) and local (Institutional Animal Care and Use Committee, Tor Vergata University, Rome, Italy) committees. C57BL/6 mice were purchased from Harlan Laboratories Srl (Urbino, Italy). 12- and 80-weeks-old mice were considered as young and old mice, respectively. Mice were fed ad libitum with standard pellet diet and free access to water. Before sacrifice mice were subjected to fasting for seven hours. Mice were killed by cervical dislocation, gastrocnemius/soleus muscle was explanted immediately, frozen on dry ice and stored −80°C.

### Cell cultures and treatments

The murine skeletal muscle C2C12 cells and human lung cancer NCI-H1299 cells were obtained from the European Collection of Cell Cultures (Salisbury, UK). NCI-H1299 cells lacking of p53 were a kind gift of Prof. Gianni Cesareni (Department Biology, University of Rome Tor Vergata). C2C12 myoblasts and NCI-H1299 cells were cultured in growth medium composed of Dulbecco's Modified Eagle's Medium (DMEM) and RPMI-1640 medium respectively, supplemented with 10% fetal bovine serum, 100 U/ml penicillin/strepto-mycin and 2mM glutamine (Lonza Sales, Basel, Switzerland) and maintained at 37°C in an atmosphere of 5% CO_2_ in air. C2C12 myoblasts were plated at 80% of confluence and cultured in growth medium for 24 h. To induce differentiation, cells were washed in PBS and growth medium was replaced with differentiation medium (DM), which contained 2% heat inactivated horse serum (Lonza, ECS0090D) [4]

BSO, a highly selective and potent inhibitor of the enzyme GCLC, was added in the culture medium at a concentration of 1 mM after 15 h from transfection. The NOS inhibitor L-NAME was used at a concentration of 100 μM (1 h before BSO treatment) and maintained throughout the experiment. The NO scavenger carboxy-PTIO was added at a concentration of 2 μM and maintained throughout the experiment as previously described [11, 27]. GSNO was added to purified nuclei at a concentration of 5 mM at 4°C for 30 min in nucleus lysis buffer (NLB) containing 50 mM Tris-HCl pH 8.1, 10 mM EDTA, 1% SDS, 10 mM sodium butyrate, protease inhibitors and incubated 1 h at 4°C.

### Transfection

C2C12 myoblasts and NCI-H1299 cells were stably transfected with the following plasmids: pcDNA3.1-p53 (Wt-p53), four mutants (single mutants: p53C124S, p53C277S, p53C275S and triple mutant C124-277-275S) or pcDNA3.1 empty vector (kindly donated by Dr. Yvonne Sun, The Cancer Institute of New Jersey, NJ and Dr. Marikki Laiho, Marikki Laiho, Haartman Institute, Department of Virology, University of Helsinki, Hensinky) by electroporation using Nucleofector 4D® (Lonza, Sales) according to the manufacturer's instructions, and were immediately seeded into fresh medium. Transfection efficiency was estimated by co-transfecting the cells with pMAX-FP-GreenC vector (Lonza Sales). Only experiments that gave transfection efficiency of 80% were considered. Twenty-four hours after transfection (day 0), differentiation was induced.

### RT-qPCR analysis

Total RNA was extracted using TRI Reagent (Sigma-Aldrich) and used for retro-transcription. qPCR was performed in triplicate by using validated qPCR primers (BLAST), Ex TAq qPCR Premix (Lonza Sales) and the Roche Real Time PCR LightCycler II (Roche Applied Science, Monza, Italy). mRNA levels were normalized to RPL, and the relative mRNA levels were determined by using the 2^−DDCt^ method [28]. The primer sequences are listed in Supplementary Table S2.

### Preparation of cell lysates and Western blot analyses

Cell pellets were resuspended in RIPA buffer (50 mM Tris-HCl, pH 8.0, 150 mM NaCl, 12 mM deoxycholic acid, 0.5% Nonidet P-40 and protease inhibitors). Protein samples were used for SDS-PAGE followed by Western blotting as previously described [12]. Nitrocellulose membranes were stained with primary antibodies against Tubulin (1:1000), PGC-1α (1:500), SOD2 (1:2000), NFE2L2 (1:1000), p53 (1:1000), p21 (1:1000), Sp1 (1:500), GSNOR (1:500), nNOS (C-terminal 1:500), Syntrophin (1:1000) and LDH (1:1000). Afterward, the membranes were incubated with the appropriate horseradish peroxidase conjugated secondary antibody, and immunoreactive bands were detected by a Fluorchem Imaging System upon staining with ECL Select Western Blotting Detection Reagent (GE Healthcare, Pittsburgh, PA, USA; RPN2235). Immunoblots reported in the figures are representative of at least four experiments that gave similar results. Tubulin and Sp1 were used as loading controls.

Proteins were assayed by the method of Lowry [29].

### Preparation of nuclear extracts

Cell pellets were resuspended in NLB. Nuclei were collected by centrifugation at 600 x g for 5 min at 4°C and pellets were resuspended in 1 ml of NLB. Subsequently, nuclei were purified on NLB containing 30% sucrose (w/v) and centrifuged at 700 x g for 10 min. The pellets were resuspended in NLB to remove nuclear debris and finally used for Western blot, oligo-pull-down or ChIP assays.

### Oligo-pull-down

The assay was performed essentially as previously described [30] by using the p53RE at −2317 and −1237 on the mouse and human PGC-1α gene promoter (*ppargc1a* and PPARGC1A), respectively (Suppl. Table S1). Briefly, nuclear protein extracts were incubated with 1 μg of promoter biotinylated at 5′ and proteins were allowed to bind the oligonucleotide for 30 min at room temperature. The oligonucleotides were precipitated with UltraLink streptavidin beads (Pierce) for 1 h at 4°C. Bound fractions were washed three times with wash buffer (20 mM Tris-HCl, pH 7.5, 1 mM EDTA, 10% glycerol, 0.1% Triton X-100), eluted with denaturing buffer, and analyzed by Western Blotting using anti-p53 antibody. Oligo-pull-down specificity was demonstrated with mutant oligonucleotides used as negative controls (data not shown).

### Chromatin immunoprecipitation assay

ChIP was carried out according to the protocol of Im et al. [31] with some modifications. Briefly, after cross-linking the nuclei extracted from C2C12 and NCI-H1299 cells were fragmented by ultrasonication using 4×15 pulse (output 10%, duty 30%). Samples were pre-cleared with pre-adsorbed salmon sperm Protein G agarose beads (1 h, 4°C), and overnight immunoprecipitation using anti-p53 or control IgG antibody was carried out. After de-cross-linking (1% SDS at 65°C for 3 h), qPCR was used to quantify the promoter binding with 30 cycles total (95°C, 1 s; 60°C, 30 s; 72°C, 60 s). Results are expressed as fold enrichment with respect to IgG control. The primers used are reported in Supplementary Table S1.

### Biotin switch assay

Biotin switch assay was performed as previously described [17]. Briefly, proteins were subjected to S-NO derivatization by incubation in the presence of ascorbate, which reduces S-NO groups. The same sample incubated in the presence of biotin without ascorbate was used as negative control. After protein separation by non-reducing SDS-PAGE and Western blot, biotinylated proteins were detected by incubation of nitrocellulose membrane with HRP-conjugated streptavidin (1:1000).

### Analysis of cell viability

Adherent (after trypsinization) and detached cells were combined, washed with PBS and directly counted by optical microscope on hemocytometer, after Trypan Blue staining.

### Statistical analysis

The results are presented as means ± S.D. Statistical evaluation was conducted by ANOVA, followed by the post Student-Newman-Keuls test. Differences were considered to be significant at p<0.05.

## SUPPLEMENTARY METHODS TABLE AND FIGURES


