# Supplementary material for: Altered S-nitrosylation of p53 is responsible for impaired antioxidant response in skeletal muscle during aging
Source: Aging (Albany NY). 2016 Dec 20;8(12):3450–62. doi: 10.18632/aging.101139 (PMC5270679; doi:10.18632/aging.101139)
Supplement: Supplementary file 1 [file aging-08-3450-s001.pdf]

SUPPLEMENTARY MATERIALS

Supplementary Table S1. List of primers used for ChIP analysis and Oligo-pull-down assay.

Genes	Sequence
mppargc1a (-2317)	FW 5'-GCGAGGTTTCTGCTTAGTCA-3'<br>RV 5'-ACAATGACTAAGCAGAAACCTCG-3'
hPPARGC1A (-1237)	FW 5'-TGTCTGTGAACTGAGGGAAAAA-3'<br>RV 5'-AGGGCTAATGCAGGTAGGTG-3'
mMuRF-1 (-351)	FW 5'-GAGCCGCGGGCGCCTCGGAAAAC-3'<br>RV 5'-AGAGCAGGCTGAGGACATGTGAAAG-3'
mAtrogin-1	Atrogin-1 agccgCATGgccaggccagatgtcc -319<br>Atrogin-1 gatcgtggcctttcaCATGtcctca +31<br>Atrogin-1 agagCAGGctgaggacatgtgaaag -40<br>Atrogin-1 acccCAAGgtccctacaagttccca -82<br>Atrogin-1 atatgaggctctggcCATGacctaa -519
hPPARGC1A (-1237)	5'-(btn)AACATGTTTATTCACACAGA-3'<br>5'-(btn)TCTGTGTGAATAAACATGTT-3'
mppargc1a (-2317)	5'-(btn)CTCTAAATAAAAAATGTTATAC-3'<br>5'-(btn)GTATAACATTTTTTATTTAGAG-3'

Supplementary Table S2. List of primers used for RT-qPCR analysis.

Genes	Sequences
mAtrogin-1 FW	5'-GCGACCTTCCCCAACGCCTG-3'
mAtrogin-1 RV	5'-GGCGACCGGGACAAGAGTGG-3'
mCGLC FW	5'-CGCACAGCGAGGAGCTTCGG-3'
mCGLC RV	5'-CTCCACTGCATGGGACATGGTGC-3'
hCGLC FW	5'-AGGAGCGAGGACTGGAGCCAT-3'
hCGLC RV	5'-GCAACATGCTGGGCCAGGAGA-3'
mMuRF-1 FW	5'-AGGGGCTACCTTCCTCTAAGTG-3'
mMuRF-1 RV	5'-TCTTCCCCAGCTGGCAGCCC-3'
mMyoD FW	5'-GGGGCCGCTGTAATCCATCATGC-3'
mMyoD RV	5'-GGAGATCCTGCGCAACGCCA-3'
mNFE2L2 FW	5'-TCCGCCAGCTACTCCCAGGTTG-3'
mNFE2L2 RV	5'-TGGGCCTGATGAGGGGCAGTG-3'
hNFE2L2 FW	5'-ACAGGAGGAGGAAGTGGAGGGACT-3'
hNFE2L2 RV	5'-TCAGCTGGCGCGTAGGTTTGT-3'
mPAX-7 FW	5'-TTCGATTAGCCGAGTGCTCA-3'
mPAX-7 RV	5'-ATCCAGACGGTTCCCTTTG-3'
mPGC-1α FW	5'-ACTGCAGGCCTAACTCCTCCCAC-3'
mPGC-1α RV	5'-CCCTCTTGGTTGGCGGTGGC-3'

hPGC-1α FW	5’-ACTGCAGGCCTAACTCCACCCA-3’
hPGC-1α RV	5’-ACTCGGATTGCTCCGGCCCT-3’
mRPL FW	5’-GTACGACCACCACCTTCCGGC-3’
mRPL RV	5’-ATGGCGGAGGGGCAGGTTCTG-3’
hRPL FW	5’-GGCGGACCGTGCGAGGTATG-3’
hRPL RV	5’-GGCGGTGGGATGCCGTCAAA-3’
mSOD2 FW	5’-GTGTCTGTGGGAGTCCAAGG-3’
mSOD2 RV	5’-AGCGGAATAAGGCCTGTTGT-3’
hSOD2 FW	5’-GCAAGGAACAACAGGCCTTA-3’
hSOD2 RV	5’-AAGAGCTTAACATACTCAGCATAAC-3’
mp21 FW	5’- CAGAATAAAAGGTGCCACAGGC-3’
mp21 RV	5’- CGTCTCCGTGACGAAGTCAA-3’

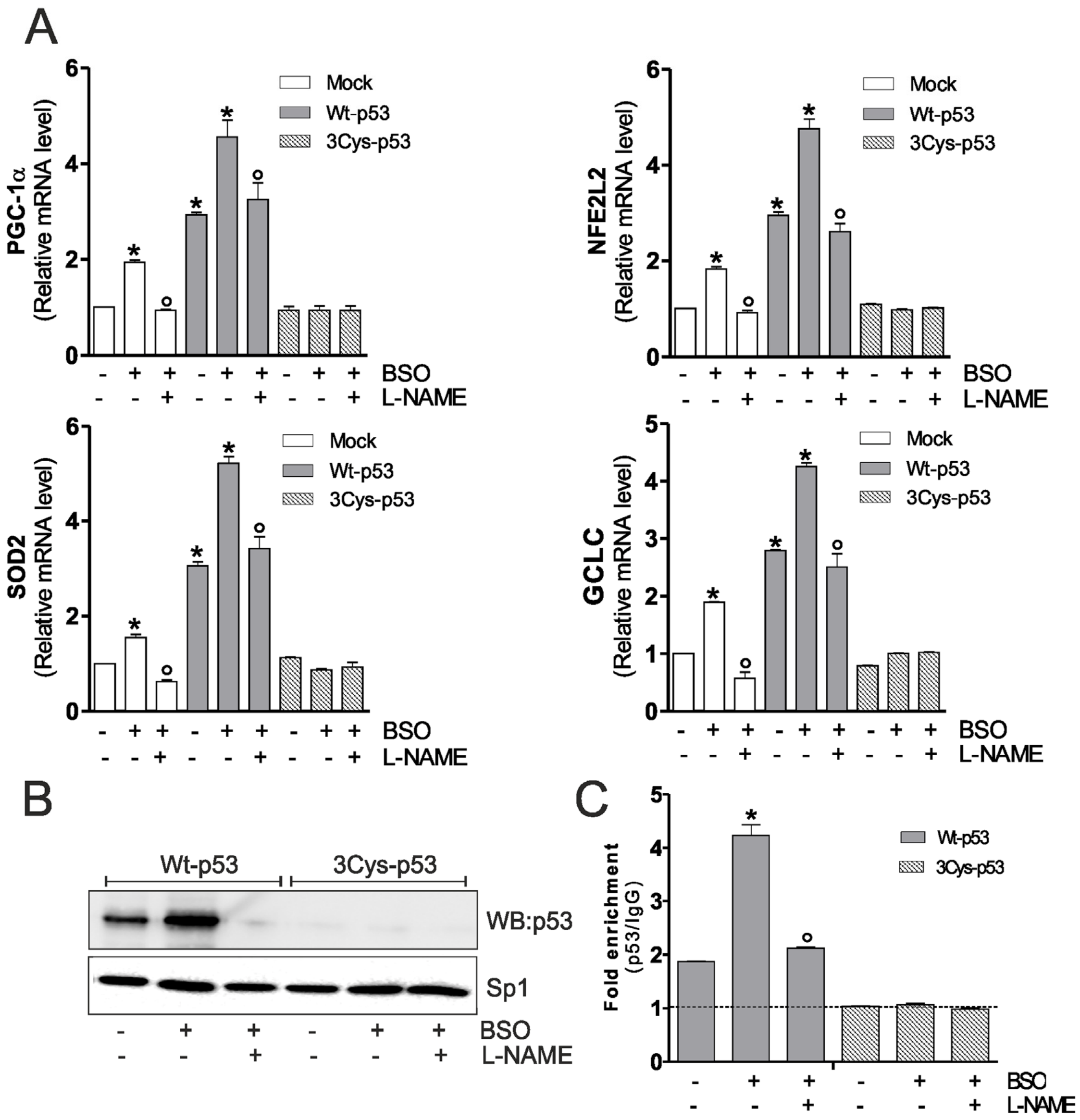

Supplementary Figure S1. 3Cys-p53 mutant impairs NO/PGC-1α-mediated antioxidant response in C2C12 myoblasts. (**A**) C2C12 myoblasts were transfected with pcDNA3.1 vector containing cDNA for wild type p53 (Wt-p53), triple p53 mutant in DBD (C277S, C275S and C124S) (3Cys-p53) or with empty vector (Mock). After 15 h from transfection myoblasts were treated with 1 mM BSO for 24 h. L-NAME (100 μM) was added 1 h before BSO treatment (15 h) and maintained throughout the experiment. Total RNA was isolated and relative mRNA levels of PGC-1α, NFE2L2, SOD2 and GCLC were analyzed by RT-qPCR. Data are expressed as means ± S.D. (n=5; *p<0.001 *vs* Mock; °p<0.01 *vs* BSO-treated cells). (**B**) Nuclear protein extracts (500 μg) were subjected to oligo-pull-down by using the biotinylated oligonucleotide representing the p53RE on the *ppargc1a* promoter and bound p53 was detected by Western blot. Twenty μg of nuclear proteins (input) were used for Western blot analysis of Sp1. (**C**) ChIP assay was carried out on cross-linked nuclei from Wt-p53 and 3Cys-p53 cells, using p53 antibody followed by qPCR analysis of p53RE. Dashed line indicates the value of IgG control. Data are expressed as means ± S.D. (n=4; *p<0.001 *vs* Mock; °p<0.001 *vs* BSO-treated cells). All the immunoblots reported are from one experiment representative of four that gave similar results.

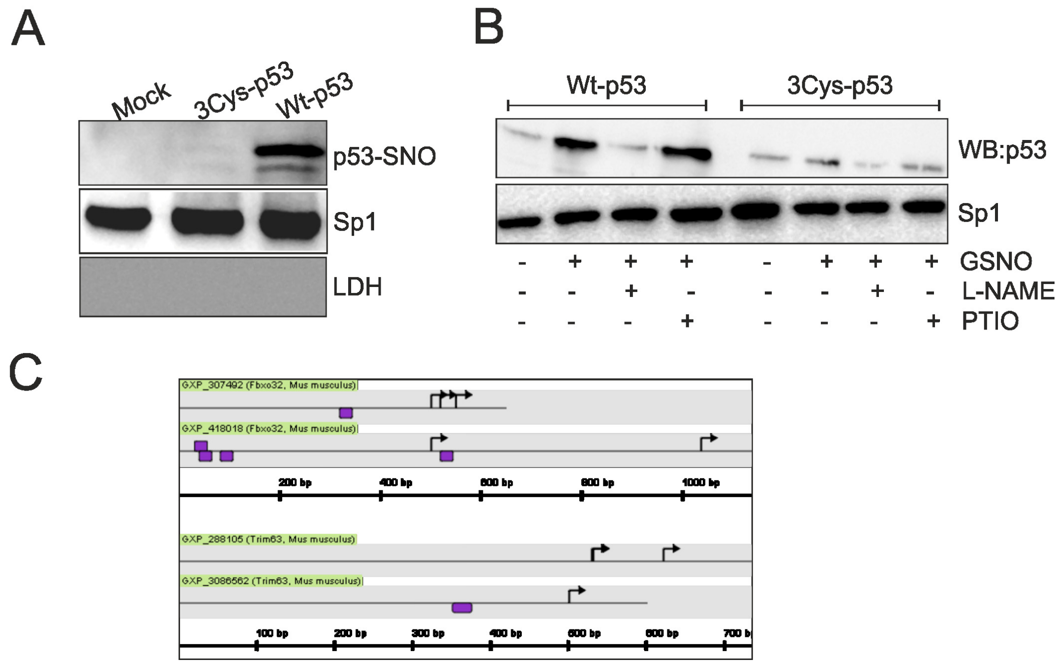

Supplementary Figure S2. 3Cys-p53 mutant not bind the p53RE on *ppargc1a* promoter. (**A**) C2C12 myoblasts were transfected with pcDNA3.1 vector containing cDNA for wild type p53 (Wt-p53), triple p53 mutant in DBD (C277S, C275S and C124S) (3Cys-p53) or with empty vector (Mock). After 15 h from transfection myoblasts were treated with 1 mM BSO for 24 h. Nuclear proteins (500 µg) were subjected to S-NO derivatization with biotin. After Western blot the nitrocellulose was incubated with p53 antibody for detection of p53-SNO. Sp1 was used as loading control. The possible presence of cytoplasmic contaminants was tested by incubating nitrocellulose with rabbit anti-LDH. (**B**) L-NAME (100 µM) was added 1 h before BSO treatment (15 h) and maintained throughout the experiment. Intact nuclei of C2C12 cells were pre-treated with 100 µM L-NAME or with 2 µM carboxy PTIO (PTIO) for 10 minute and subsequently incubated with 5 mM GSNO at 4°C for 30 minute. Nuclear protein extracts (500 µg) were subjected to oligo-pull-down by using the biotinylated oligonucleotide representing the p53RE on the *ppargc1a* promoter and bound p53 was detected by Western blot. Twenty µg of nuclear proteins (input) were used for Western blot analysis of Sp1. (**C**) Schematic representation of murine Atrogin-1 (Fbox32, *upper*) and MuRF-1 (Trim63, *bottom*) promoters. The black arrows indicate the p53RE identified on Atrogin-1 and MuRF-1 promoters. All the immunoblots reported are from one experiment representative of four that gave similar results.
